# Antibiofilm Effect of Nitric Acid-Functionalized Carbon Nanotube-Based Surfaces against *E. coli* and *S. aureus*

**DOI:** 10.3390/antibiotics12111620

**Published:** 2023-11-11

**Authors:** Marisa Gomes, Rita Teixeira-Santos, Luciana C. Gomes, Francisca Sousa-Cardoso, Fábio M. Carvalho, Andreia R. Tomé, Olívia S. G. P. Soares, Kathryn A. Whitehead, Filipe J. Mergulhão

**Affiliations:** 1 LEPABE—Laboratory for Process Engineering, Environment, Biotechnology and Energy, Faculty of Engineering, University of Porto, Rua Dr. Roberto Frias, 4200-465 Porto, Portugal; marisagomes@fe.up.pt (M.G.); ritadtsantos@fe.up.pt (R.T.-S.); luciana.gomes@fe.up.pt (L.C.G.); mfcardoso@fe.up.pt (F.S.-C.); up201502963@edu.fe.up.pt (F.M.C.); up201806129@edu.fe.up.pt (A.R.T.); 2 ALiCE—Associate Laboratory in Chemical Engineering, Faculty of Engineering, University of Porto, Rua Dr. Roberto Frias, 4200-465 Porto, Portugal; salome.soares@fe.up.pt; 3 LSRE-LCM—Laboratory of Separation and Reaction Engineering, Laboratory of Catalysis and Materials, Faculty of Engineering, University of Porto, Rua Dr. Roberto Frias, 4200-465 Porto, Portugal; 4 Microbiology at Interfaces Group, Manchester Metropolitan University, Manchester M1 5GD, UK; k.a.whitehead@mmu.ac.uk

**Keywords:** modified carbon nanotubes, poly(dimethylsiloxane) composites, *Escherichia coli*, *Staphylococcus aureus*, antimicrobial activity, medical devices

## Abstract

Chemically modified carbon nanotubes are recognized as effective materials for tackling bacterial infections. In this study, pristine multi-walled carbon nanotubes (p-MWCNTs) were functionalized with nitric acid (f-MWCNTs), followed by thermal treatment at 600 °C, and incorporated into a poly(dimethylsiloxane) (PDMS) matrix. The materials’ textural properties were evaluated, and the roughness and morphology of MWCNT/PDMS composites were assessed using optical profilometry and scanning electron microscopy, respectively. The antibiofilm activity of MWCNT/PDMS surfaces was determined by quantifying culturable *Escherichia coli* and *Staphylococcus aureus* after 24 h of biofilm formation. Additionally, the antibacterial mechanisms of MWCNT materials were identified by flow cytometry, and the cytotoxicity of MWCNT/PDMS composites was tested against human kidney (HK-2) cells. The results revealed that the antimicrobial activity of MWCNTs incorporated into a PDMS matrix can be efficiently tailored through nitric acid functionalization, and it can be increased by up to 49% in the absence of surface carboxylic groups in f-MWCNT samples heated at 600 °C and the presence of redox activity of carbonyl groups. MWCNT materials changed the membrane permeability of both Gram-negative and Gram-positive bacteria, while they only induced the production of ROS in Gram-positive bacteria. Furthermore, the synthesized composites did not impact HK-2 cell viability, confirming the biocompatibility of MWCNT composites.

## 1. Introduction

The insertion or implantation of medical devices into the human body has become a recurrent strategy to replace or even repair damaged organs [1,2]. In fact, it is estimated that over 5 million medical devices, including cardiac implantable devices, urinary or central venous catheters, orthodontic and orthopedic implants, ocular prostheses and contact lenses, hemodialyzers, and intrauterine contraceptive devices, are used each year in the United States alone [3]. Although these devices have revolutionized the medical field, being essential for the prevention, diagnosis, treatment, and rehabilitation of many illnesses and diseases, they are also strongly associated with an increased risk of opportunistic infection [4,5]. Medical device-associated infections (DAIs), whose incidence depends on the type and physicochemical properties of the prosthetic device or implant, the local hydrodynamic conditions, the microorganisms involved, and indwelling time [6,7], are recognized as a significant public health challenge [8]. According to the Centers for Disease Control, DAIs represent approximately 50 to 70% of the nearly two million healthcare-related infections reported annually [9,10]. The associated mortality rates, which can range from <5% for devices such as bladder catheters, fracture fixation devices, joint prostheses, and dental implants, to >25% for mechanical heart valves and heart assist devices [11], as well as the imposed financial burden (more than USD 3 billion spent annually in the United States and GBP 7 million in the United Kingdom [12,13]), reinforce the seriousness of the problem.

The biofilm mode of growth adopted by the infectious agents and the increased resistance to conventional antimicrobial strategies are the main factors that interfere with the extent and severity of DAIs [14]. When a well-organized and structured biofilm is formed, its exopolysaccharide matrix acts as a barrier, protecting the cells from host defense mechanisms and antimicrobial agents and contributing to the chronic nature of such infections [15,16,17]. Consequently, implant removal or replacement, along with long-term antibiotic therapy, is often the only possible option to overcome the infection [18]. These limitations have, therefore, motivated the search for new engineered implant surfaces that could hamper microbial colonization and biofilm formation. Some of the proposed strategies for manufacturing medical devices include bacteria-repelling and anti-adhesive surfaces, antibacterial drug-release coatings, nanostructured coatings, and coatings containing bioactive molecules capable of destroying pre-established biofilms [18,19,20,21]. Although most of these approaches are still far from being used in clinical practice, an increasing number of studies exploring the use of carbon nanotubes (CNTs) as alternative materials can be found.

CNTs consist of hollow and concentric cylindrical tubes formed by rolled graphene sheets, and their functionalization with drugs, antimicrobial peptides, enzymes, metals, polymers, or photosensitizers has been extensively studied and implemented to produce effective antimicrobial surfaces for application in biomedical devices and prosthetic implants [22,23]. Although the CNT mode of action is not fully characterized, in general, CNTs can induce antimicrobial effects by (i) destroying the microorganisms’ cell membranes through direct piercing; (ii) attaching to the microbial cell surface to promote transmembrane electron transfer, consequently causing cell wall and membrane destruction; (iii) inducing protein dysfunction and DNA damage; and (iv) generating secondary products, such as reactive oxygen species (ROS). The overall antimicrobial and antiadhesive performance of CNT derivatives depends on multiple factors, including CNT type, single- (SWCNT) or multi-walled (MWCNTs), length, purity, and electronic structure [23,24,25,26]. The functionalization of CNTs with acid/carboxylic moieties has also been shown to interfere with their antimicrobial activity. Over the last years, a growing number of studies has reported the greater efficacy of carboxylated MWCNTs against common infectious agents [25,26,27,28,29,30,31,32,33]. However, to the best of our knowledge, the application of these acid-functionalized CNTs as coatings for medical devices is still poorly documented, with data from only two studies [31,32]. Furthermore, there is a lack of research on the effectiveness of oxidized MWCNTs with different acidic levels in inhibiting microbial growth [34,35].

This study aimed to investigate the antibiofilm activity of nitric acid-functionalized MWCNT-based surfaces against two common colonizers of medical devices—*Escherichia coli* and *Staphylococcus aureus*. MWCNTs with different acidic levels were obtained via oxidation treatment with nitric acid, followed by a temperature-selective reduction of surface oxygen functional groups. Additionally, the main mechanisms behind the antimicrobial activity of the functionalized MWCNTs were assessed, thereby contributing to a deeper understanding of the interactions that occur at the interface between bacterial cells and MWCNT nanocomposites. Furthermore, the cytotoxicity of produced composites was assessed on a human cell line.

## 2. Results and Discussion

Considering the potential of CNT surface modification in enhancing its antimicrobial activity, polydimethylsiloxane (PDMS) surfaces containing nitric acid-functionalized MWCNT materials were produced and characterized by optical profilometry and scanning electron microscopy (SEM). Subsequently, their antibiofilm performance was evaluated against pure cultures of *E. coli* and *S. aureus*, and their effect on the viability of human kidney cells was tested.

### 2.1. Characterization of MWCNTs

**Figure 1:**
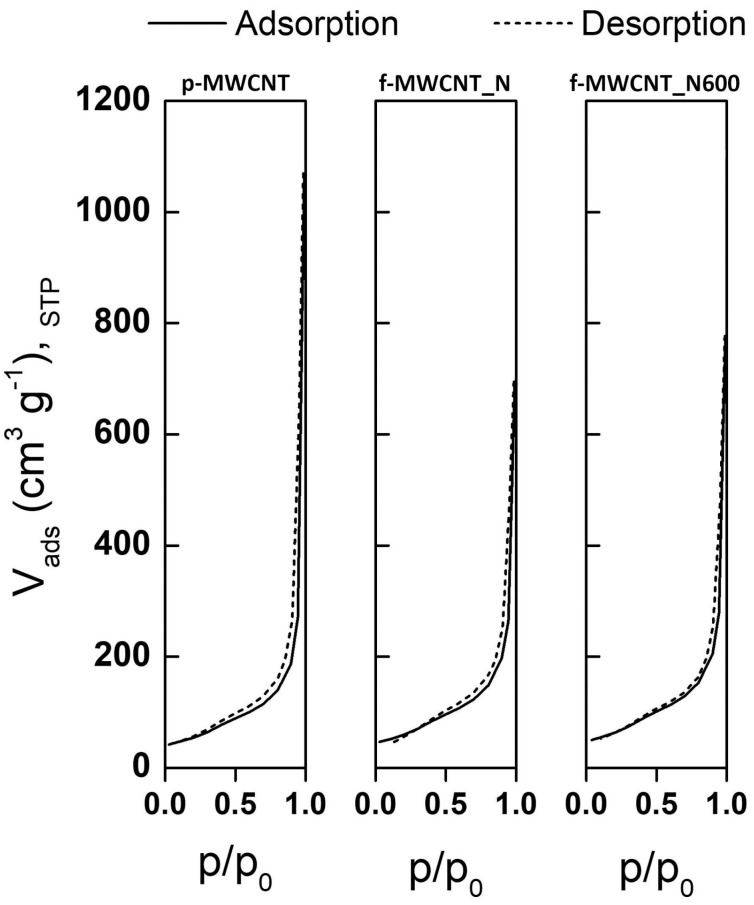
N_2_ adsorption–desorption isotherms at −196 °C for pristine multi-walled carbon nanotubes (p-MWCNT), nitric acid-functionalized multi-walled carbon nanotubes (f-MWCNT_N), and f-MWCNT_N heated at 600 °C (f-MWCNT_N600).

To evaluate the textural modifications introduced during the chemical and thermal treatments of MWCNTs, the N_2_ adsorption–desorption isotherms of pristine MWCNTs (p-MWCNT), nitric acid-functionalized MWCNTs (f-MWCNT_N), and f-MWCNT_N samples heated at 600 °C (f-MWCNT_N600) were determined (Figure 1).

According to IUPAC classification, the isotherms of both the original and modified MWCNTs fit the Type IV isotherm profile, since they are characterized by the presence of a hysteresis loop, which is associated with capillary condensation occurring in mesopores, and the limiting uptake over a range of high p/p_0_ [36]. Although some authors have shown that N_2_ adsorption isotherms of nitric acid-functionalized MWCNT samples can be classified as type II (typical of non-porous materials, according to IUPAC) [37,38,39,40], the physisorption isotherms obtained in the present work are in agreement with previous results on pristine and nitric acid-functionalized MWCNT samples with similar synthesis procedures, average diameter and length, and purity [41,42]. Despite the similar shapes of the isotherms, it is possible to observe some differences regarding the amount of N_2_ adsorbed at high relative pressure (as demonstrated by the V_p_ at p/p_0_ = 0.95) and the S_BET_ values shown in Table 1.

Although no relevant changes were observed in the S_BET_ values, an increase in the surface area of oxidized MWCNT samples compared to p-MWCNTs was obtained. According to previous studies, this increase can be explained by the opening of MWCNT end caps and the creation of sidewall openings, which are structural defects that increase the available area for N_2_ adsorption [37,40,43,44]. With the thermal treatment at 600 °C, part of the chemical groups introduced during oxidation treatment with HNO_3_ were removed, leading to a slight increase in the specific surface area [37]. Contrary to that observed for S_BET_, chemical functionalization with nitric acid resulted in a decrease in the V_p_ from 0.419 to 0.408 cm^3^ g^−1^ for p-MWCNT and f-MWCNT_N samples, respectively. This decrease in the V_p_ is in accordance with the literature on nitric acid-treated MWCNT samples [38,39,41,44] and may be related to the different extent of CNT agglomeration, which is typical of samples with different surface chemistries [40,45]. In turn, the heat treatment at 600 °C resulted in an increase in V_p_ (from 0.408 to 0.432 cm^3^ g^−1^, when f-MWCNT_N and f-MWCNT_N600 are compared), which is in agreement with previously published results [40]. Taken together, these results suggest that the chemical modification of MWCNTs’ surface was successfully accomplished. In general, considering that higher surface areas potentiate the interaction between MWCNTs and bacterial cells and, consequently, cell death [46], nitric acid-functionalized MWCNTs are expected to be promising materials for the development of new antimicrobial coatings.

### 2.2. Characterization of MWCNT/PDMS Surfaces

**Figure 2:**
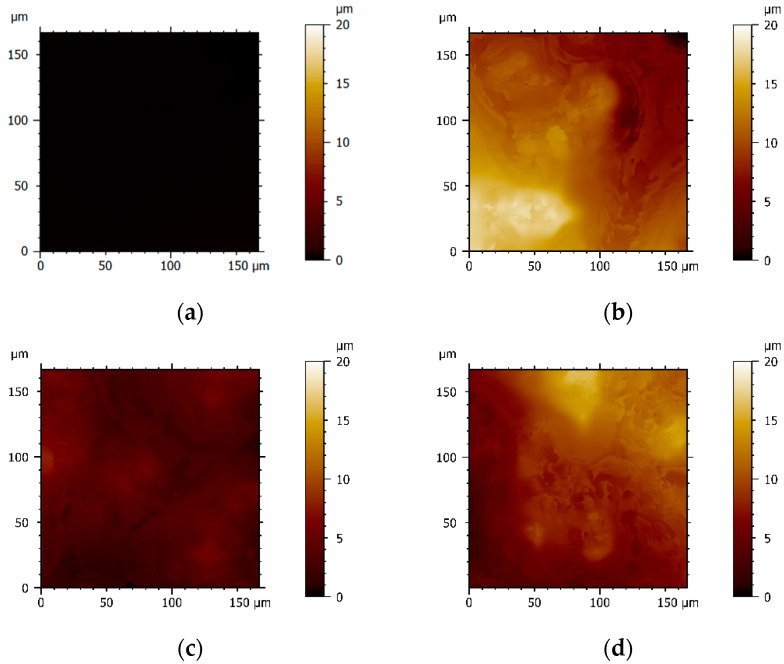
Representative 2D profilometry images of (**a**) PDMS, (**b**) p-MWCNT/PDMS, (**c**) f-MWCNT_N/PDMS, and (**d**) f-MWCNT_N600/PDMS (scan area of 170 × 170 µm). The vertical color bars represent the range of surface heights (*z*-range) of the images.

**Figure 3:**
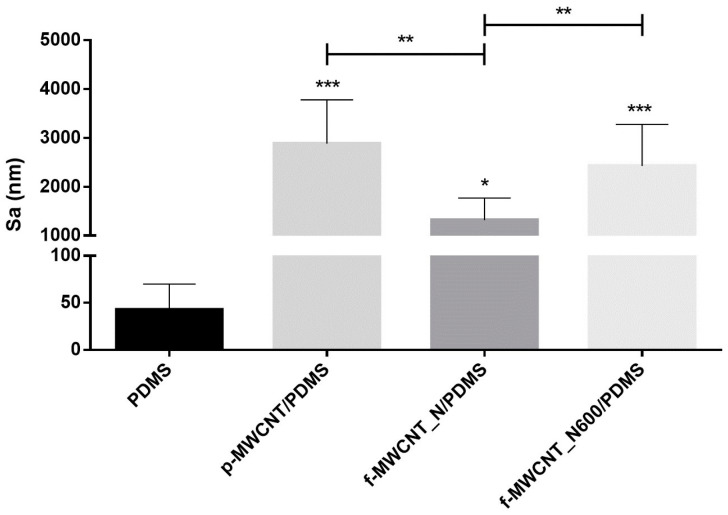
Mean roughness (*Sa*) of PDMS (control) and MWCNT-based surfaces. The means ± standard deviations are presented. Significant differences are indicated as * (*p* < 0.05), ** (*p* < 0.01), and *** (*p* < 0.001).

Surface topography is known to influence the extent of cell attachment and, consequently, the formation of biofilms [47,48]. In the present work, optical profilometry was used to characterize the roughness of PDMS, p-MWCNT/PDMS, f-MWCNT_N/PDMS, and f-MWCNT_N600/PDMS composites. As shown in the profilometry images (Figure 2), the PDMS coatings resulted in a surface without discernible topographies and irregularities distinguishable on the micrometric scale (Figure 2a). With the addition of MWCNTs, an increase in surface roughness, characterized by the presence of irregular peaks and features, was observed (Figure 2b–d). This propensity for CNTs to form aggregates and accumulate in the peaks, increasing the overall surface roughness, has been described in previous studies [49,50]. By comparing the topographic images of p-MWCNT/PDMS and f-MWCNT_N/PDMS (Figure 2b and Figure 2c, respectively), it was observed that a more homogeneous dispersion and distribution of f-MWCNT_N into the PDMS matrix was obtained. This observation was confirmed by determining the arithmetical mean height (*Sa*) of the assessed surface profiles (Figure 3). The surfaces containing p-MWCNT, f-MWCNT_N, or f-MWCNT_N600 exhibited significantly increased *Sa* values when compared with PDMS (*p* < 0.05). Additionally, when comparing p-MWCNT/PDMS with f-MWCNT_N/PDMS surfaces, a significant reduction in the mean roughness from 2.9 to 1.3 µm was observed (*p* < 0.01, Figure 3). These results are supported by previous studies that showed that by promoting a stronger interfacial bonding between CNTs and the polymer matrix, acid functionalization can reduce the tendency of CNTs to form aggregates and consequently reduce the overall surface roughness [51,52,53]. Moreover, the introduction of oxygen-containing groups is recognized for reducing the entanglement degree of the CNTs, enhancing their dispersion within the polymeric matrix [54]. The surface roughness of the composites containing f-MWCNT_N tended to increase again after heat treatment (Figure 2d). Indeed, when comparing f-MWCNT_N/PDMS with f-MWCNT_N600/PDMS composites, a significant increase in the average roughness from 1.3 to 2.4 µm was observed (*p* < 0.01, Figure 3). This result might be explained by the heating process eroding the surface of the f-MWCNT_N600/PDMS surface, thus making it more topographically similar to the p-MWCNT/PDMS surface, but further investigation is required.

**Figure 4:**
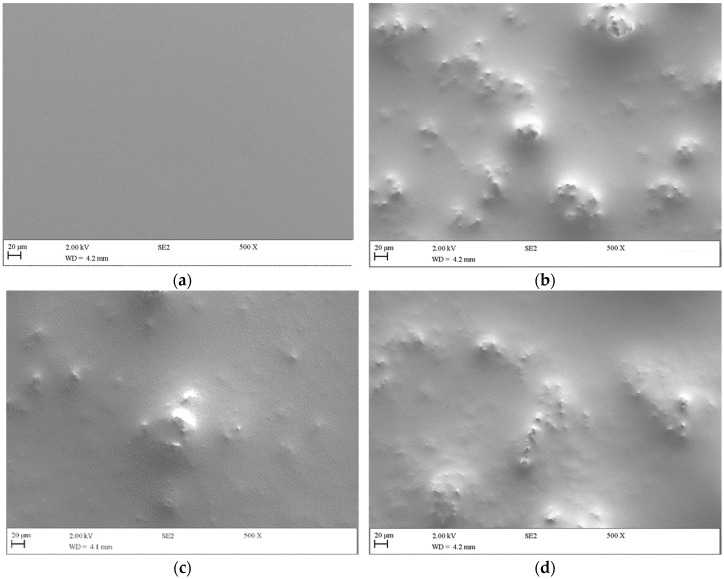
SEM images of (**a**) PDMS, (**b**) p-MWCNT/PDMS, (**c**) f-MWCNT_N/PDMS, and (**d**) f-MWCNT_N600/PDMS composites (magnification of 500×).

The roughness characterization of MWCNT/PDMS composites was complemented by SEM analysis (Figure 4 and Figure S1 in Supplementary Material). SEM images of the composites revealed the presence of several MWCNT agglomerates on their surfaces (Figure 4b–d) compared to bare PDMS (Figure 4a). Representative micrographs of p-MWCNT/PDMS and f-MWCNT_N/PDMS cross-sections also confirmed the dispersion of nanotube agglomerates through the surfaces (Figure S2 in Supplementary Material). The f-MWCNT_N/PDMS surface (Figure 4c) had less visually protruding clusters than p-MWCNT/PDMS (Figure 4b). However, the number of clusters on the p-MWCNT/PDMS and f-MWCNT_N600/PDMS composites was visually similar (Figure 4b and Figure 4d, respectively). With thermal treatment, f-MWCNT_N tends to decrease the number of oxygen-containing groups on the surface, and this may lead to a decrease in its dispersion into the polymeric matrix. These results correlate with the optical profilometry data.

### 2.3. Antibiofilm Analysis

**Figure 5:**
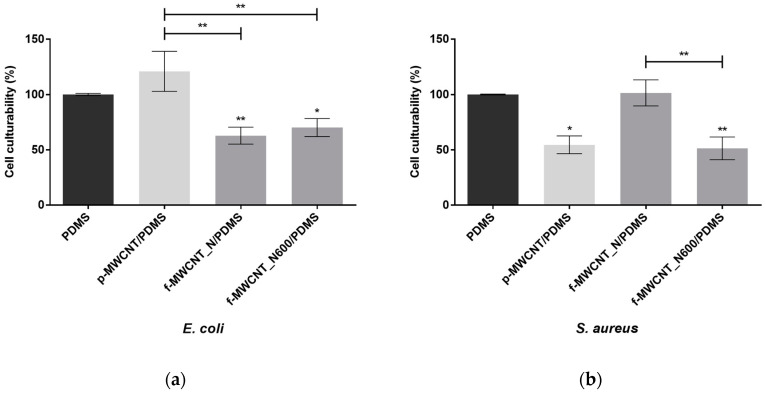
Antibiofilm performance of bare PDMS (control, 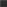), p-MWCNT/PDMS (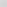), and f-MWCNT_N-based surfaces (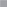) against (**a**) *E. coli* and (**b**) *S. aureus*. The means ± standard error for at least three independent experiments are represented. Significant differences are indicated as * (*p* < 0.05) and ** (*p* < 0.01).

Several surface properties, such as surface charge and composition, are known to affect protein adsorption, and consequently, bacterial attachment and proliferation [55]. In the present study, the influence of MWCNT surface chemistry on the antibiofilm activity of MWCNT-based surfaces against *E. coli* and *S. aureus* was evaluated. Figure 5 presents the percentage of biofilm cell culturability after 24 h of growth on bare PDMS, p-MWCNT/PDMS, f-MWCNT_N/PDMS, and f-MWCNT_N600/PDMS surfaces. Considering that no MWCNT leaching was observed (UV-Vis spectra as Figure S3 in Supplementary Material), it is reasonable to assume that the achieved data are a result of the action of the MWCNTs embedded into the PDMS matrix. Regarding *E. coli* biofilm growth, the p-MWCNT/PDMS surfaces had no significant effect on biofilm cell culturability (*p* > 0.05, Figure 5a). This result is in accordance with a previous study in which no significant differences were observed in the number of culturable cells following *E. coli* biofilm growth on composite surfaces with the same MWCNT loading [50]. As for the f-MWCNT_N/PDMS surface, characterized by the strongest acidic character, a significant reduction in the number of *E. coli* culturable cells compared to bare PDMS was determined (~37%, *p* < 0.01). Previous studies have indicated that the introduction of functional oxygen-containing groups on the surface of MWCNTs increased their hydrophilicity and ability to interact with microbial cells, thus promoting cell death [33,56,57]. Additionally, the thermally treated f-MWCNT_N600/PDMS composite promoted a significant reduction in the number of *E. coli* biofilm culturable cells (~30% compared with bare PDMS; *p* < 0.05). However, there were no significant differences in the cell culturability of biofilms growing on f-MWCNT_N/PDMS and f-MWCNT_N600/PDMS (*p* > 0.05).

When considering the biofilm growth of *S. aureus*, the p-MWCNT/PDMS surfaces significantly reduced the number of culturable cells by ~45%, compared with bare PDMS (*p* < 0.05, Figure 5b). The f-MWCNT_N/PDMS surfaces had no effect on *S. aureus* biofilm cell culturability, which is in contrast to the findings demonstrated for *E. coli*. However, several authors have demonstrated the antimicrobial potential of oxidized MWCNTs against *S. aureus* [27,28,29,30]. In addition, the discrepancy in biofilm formation by *E. coli* (Gram-negative and rod-shaped bacteria) and *S. aureus* (Gram-positive and spherical-shaped bacteria) on p-MWCNT/PDMS and f-MWCNT_N/PDMS surfaces can be explained by the differences in bacteria cell wall composition. Gram-positive bacteria feature a cytoplasmic membrane enclosed by a peptidoglycan layer, while Gram-negative bacteria have a more intricate cell envelope comprising a plasma membrane, a peptidoglycan cell wall, and an outer membrane mainly composed of lipopolysaccharide [33].

The thermally treated f-MWCNT_N600/PDMS surface significantly reduced the percentage of *S. aureus* biofilm culturable cells by 49% compared to the bare PDMS (*p* < 0.01; Figure 5b). This result has been corroborated by studies which suggest that heat treatment at 600 °C has a direct impact on the antimicrobial performance of oxidized MWCNTs. The absence of carboxylic groups on f-MWCNT_N600 samples and the redox activity of carbonyl groups has been demonstrated to potentiate CNT antimicrobial activity [34,35].

In general, it can be assumed that the antimicrobial activity of p-MWCNT/PDMS, f-MWCNT_N/PDMS, and f-MWCNT_N600/PDMS is a result of the combination of different intrinsic and extrinsic factors, including surface chemistry, roughness, and bacterial properties (cell size and shape), which are known to ultimately influence *E. coli* and *S. aureus* colonization patterns [33,58,59].

### 2.4. MWCNT Antibacterial Mechanisms

**Figure 6:**
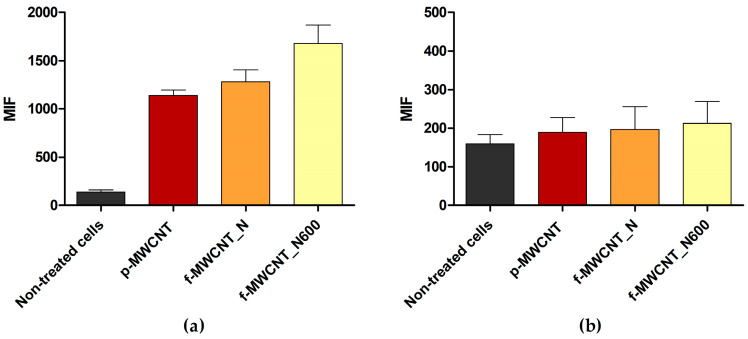
Mean intensity of fluorescence (MIF) of *E. coli* non-treated and treated with 5% (*w*/*v*) p-MWCNT, f-MWCNT_N, and f-MWCNT_N600, stained with (**a**) DiBAC_4_(3) and (**b**) DCFH-DA, respectively.

**Figure 7:**
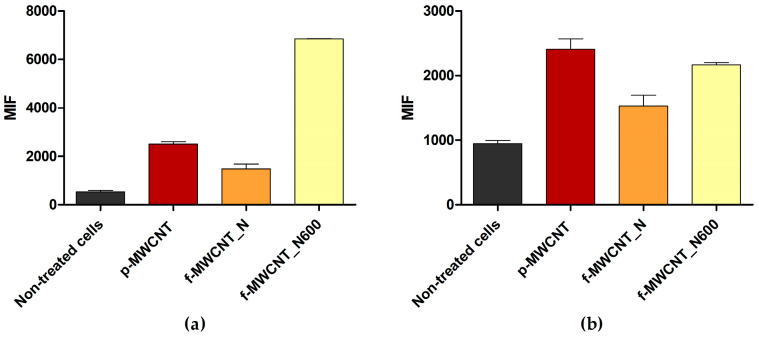
Mean intensity of fluorescence (MIF) of *S. aureus* non-treated and treated with 5% (*w*/*v*) p-MWCNT, f-MWCNT_N, and f-MWCNT_N600, stained with (**a**) DiBAC_4_(3) and (**b**) DCFH-DA, respectively.

Several studies have demonstrated the antibacterial activity of MWCNT materials against Gram-negative and Gram-positive bacteria [22,50]. However, their mechanisms of action need further clarification. To assess the antibacterial mechanisms of p-MWCNT, f-MWCNT_N, and f-MWCNT_N600, *E. coli* and *S. aureus* were exposed to 5% (*w*/*v*) MWCNT for 24 h and stained with bis-(1,3-dibutylbarbituric acid) trimethine oxonol (DiBAC_4_(3), a membrane depolarization marker) and 2′,7′-dichlorofluorescein diacetate (DCFH-DA, an ROS production indicator). The samples were then analyzed using flow cytometry (Figure 6 and Figure 7).

Flow cytometric data suggested that under the tested conditions, p-MWCNT, f-MWCNT_N, and f-MWCNT_N600 induced changes in the cell membrane permeability of *E. coli* (Figure 6a) and *S. aureus* (Figure 7a), as evidenced by the increase in the mean intensity of fluorescence (MIF) in cells treated with MWCNTs compared to untreated cells.

For *E. coli*, this effect was more pronounced in cells exposed to f-MWCNT_N and f-MWCNT_N600, which displayed a 9.1- and 11.9-fold higher MIF than the control (non-treated cells; Figure 6a). For *S. aureus*, the material which caused greater cell membrane perturbation was f-MWCNT_N600, with cells exhibiting a 12.9-fold higher MIF compared to the control (non-treated cells) and a 4.6-fold higher MIF compared to cells treated with f-MWCNT_N (Figure 7a). These results are consistent with the findings from biofilm culturability analysis and with literature reports which suggest that the antimicrobial effect of MWCNT is associated with bacterial membrane disruption [60] and that MWCNT oxidation increases its antimicrobial performance [34,35].

Furthermore, the results indicated that ROS production occurred in *S. aureus* cells exposed to MWCNTs (Figure 7b). This effect was more obvious upon exposure to p-MWCNTs and f-MWCNTs_N600, with cells presenting a 2.5- and 2.3-fold higher MIF than the control, respectively. In addition, there was a 1.4-fold decrease in MIF value when comparing f-MWCNT_N600 and f-MWCNT_N. In contrast, under the tested conditions, MWCNT exposure did not seem to induce ROS production in *E. coli* cells (Figure 6b), as there were no differences in MIF between treated and non-treated cells. Although some authors have reported that exposure to MWCNTs triggers ROS production [22,60], in this study, this effect was only detected in Gram-positive bacteria. This difference may be attributed to the different morphologies of Gram-positive and Gram-negative bacteria. In Gram-negative bacteria, the outer membrane acts as a barrier that confers resistance to external stress [61]. This feature is likely to limit the penetration of MWCNT materials, which induce ROS production in bacterial cells. This emphasizes that MWCNTs antimicrobial activity depends not only on their chemical and structural properties but also on the characteristics of the bacteria with which they interact.

### 2.5. Effect of MWCNT/PDMS on Human Cell Viability

The cytotoxicity of MWCNT/PDMS composites towards HK-2 cells was investigated to evaluate the potential adverse effects of carbon materials on renal/urinary cells (Figure S4 in Supplementary Material). HK-2 cells were cultured on MWCNT/PDMS surfaces for 24 and 48 h, followed by an assessment of cell viability. After 24 h of incubation, there was a 38% increase in cell viability on MWCNT/PDMS surfaces compared to the PDMS control. After 48 h of incubation, a slight decrease in cellular viability (approximately 13%) was observed. These results demonstrate the short-term biocompatibility of MWCNT/PDMS composites with HK-2 cells, thereby supporting their application in biomedical devices intended for short-term use. However, additional research is needed to comprehensively assess the long-term effect of carbon nanotube composites on human cell behavior.

## 3. Materials and Methods

### 3.1. Functionalization of Pristine MWCNTs

**Figure 8:**
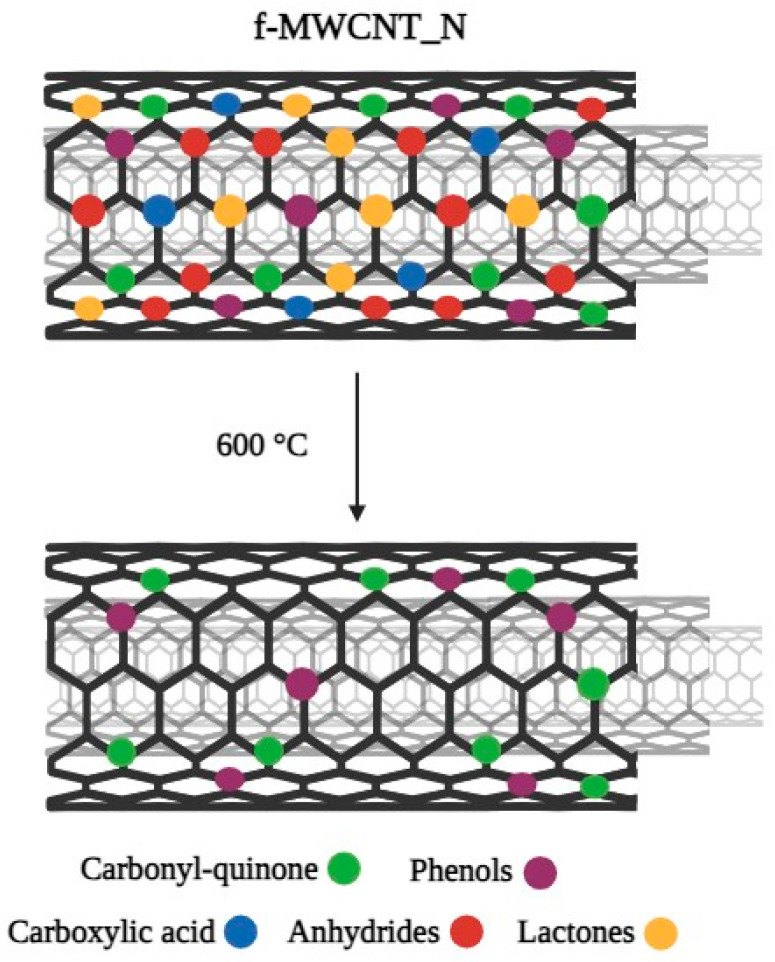
Schematic representation of the chemical groups released from the surface of nitric acid-functionalized multi-walled carbon nanotubes (f-MWCNT_N) during thermal treatment. As the temperature increased, the oxygen-containing functional groups were gradually removed. After heat treatment at 600 °C, the f-MWCNT_N600 surface contained only carbonyl-quinone and phenol groups based on [62].

Pristine MWCNTs (p-MWCNTs, Nanocyl™ NC3100, Sambreville, Belgium) produced by catalytic chemical vapor decomposition (1.5 μm average length, 9.5 nm average diameter, and more than 95% carbon purity) were used. To understand the influence of different surface modifications on the antibiofilm performance of carbon-based composites, MWCNTs were subjected to an oxidation treatment with nitric acid (HNO_3_, Merck KGaA, Darmstadt, Germany), followed by the selective removal of oxygen-containing functional groups through heat treatment at 600 °C (Figure 8) [37,40]. Briefly, functionalized MWCNTs with the strongest acid character and the highest amount of O-containing surface groups (f-MWCNT_N) were obtained by oxidizing 4 g of p-MWCNTs with 300 mL HNO_3_ 7 M in liquid phase at boiling temperature for 3 h (in a round-bottom flask connected to a condenser). Subsequently, the oxidized MWCNTs were washed with distilled water until they reached a neutral pH and then dried overnight at 110 °C. In order to produce MWCNT samples with reduced acid character by selectively removing O-containing groups, the resulting f-MWCNT_N sample was heated under an N_2_ inert atmosphere (100 cm^3^ min^−1^) at 10 °C min^−1^ heating rate, until 600 °C, where it was kept for 1 h [45]. This sample was designated as f-MWCNT_N600.

In a previous study [62], p-MWCNTs, f-MWCNT_N, and f-MWCNT_N600 samples were characterized by temperature-programmed desorption mass spectrometry. This analysis demonstrated that HNO_3_ oxidation results in the incorporation of a large amount of O-containing groups onto the MWCNT surface, while the thermal treatment at 600 °C removes carboxylic acids, anhydrides, and lactones (Figure 8).

### 3.2. Synthesis of MWCNT/PDMS Surfaces

MWCNT-based composites were produced through a bulk mixing process, as previously described [26]. Firstly, p-MWCNT, f-MWCNT_N, and f-MWCNT_N600 were incorporated into a PDMS matrix (Sylgard 184 Part A, Dow Corning, Midland, MI, USA) at a loading of 5 wt.% MWCNT. PDMS was selected as the polymeric matrix due to its widespread use in the fabrication of medical devices and implants [63]. After stirring for 30 min at 500 rpm, the mixture was submitted to an ultrasonic treatment using a Hielscher UP400S device (at 200 W and 12 kHz) for 60 min. Subsequently, it was placed in an ultrasonic bath (Ultrasons 3000514, JP Selecta, Barcelona, Spain) for an additional 30 min to remove any entrapped air bubbles and facilitate the spreading of MWCNT. The curing agent (Sylgard 184 Part B, Dow Corning, Midland, MI, USA) was added in an A:B ratio of 10:1, and the resulting mixture was properly homogenized. Thin films of PDMS (control surface) and each nanocomposite material (p-MWCNT/PDMS, f-MWCNT_N/PDMS, and f-MWCNT_N600/PDMS) were formed on top of small glass coupons using the spin coating technique (Spin150-v3.2, APT GmbH, Bienenbüttel, Germany) under predefined conditions (6000 rpm for 1 min). The coated coupons were heated at 80 °C overnight to facilitate the curing process [50].

### 3.3. Textural Characterization of MWCNTs

The textural characterization of p-MWCNT, f-MWCNT_N, and f-MWCNT_N600 samples was based on the N_2_ adsorption isotherms, obtained at −196 °C in a Quantachrome NOVA 4200e multi-station apparatus (Quantachrome Instruments, Boynton Beach, FL, USA). Prior to the analysis, the samples were degassed at 150 °C for 3 h under vacuum. The Brunauer–Emmett–Teller specific surface area (S_BET_) was calculated from the N_2_ adsorption curve in the relative pressure range of 0.05–0.3, while the total pore volume (V_p_) was determined from the amount adsorbed at p/p_0_ = 0.95 [40].

### 3.4. Characterization of MWCNT/PDMS Surfaces

#### 3.4.1. MWCNT/PDMS Surface Topography

Optical profilometry was used to assess the surface topography of PDMS, p-MWCNT/PDMS, f-MWCNT_N/PDMS, and f-MWCNT_N600/PDMS composites. The analysis was performed using a MicroXAM surface mapping microscope (ADE Corporation, XYZ model 4400 mL system, Tucson, AZ, USA) coupled to an AD phase-shift controller (Omniscan, Wrexham, UK). Three different areas of three coupons of each surface type were imaged (n = 9), and the MAPVIEW AE 2.17 (Omniscan, Wrexham, UK) image analysis system was used to obtain the mean roughness parameter ($S_{a}$). Two-dimensional (2D) profilometry images were extracted using MountainsMap^®^ Imaging Topography software (version 10.0.10433; Digital Surf, Besançon, France).

#### 3.4.2. MWCNT/PDMS Surface Morphology

The surface morphology of the produced nanocomposites was assessed by scanning electron microscopy (SEM) using a Zeiss Supra 40VP field emission gun scanning electron microscope (Carl Zeiss Ltd., Cambridge, UK). The surfaces were initially fixed to SEM stubs using a conductive double-sided adhesive pad (Agar Scientific, Stansted, UK) and sputtered with gold for 30 s using an SEM coating system (Polaron, London, UK). A secondary electron detector was used to obtain surface and cross-section images of the composites.

### 3.5. Evaluation of MWCNT Leaching from PDMS Surfaces

Ultraviolet–Visible (UV-Vis) spectroscopy was used to evaluate the release of MWCNTs from the PDMS matrix. The coated coupons were immersed in 3 mL of sterile distilled water, and the samples were incubated at 37 °C for 24 h with agitation [64]. The liquid was removed, filtered with 0.2 μm sterile syringe filters (Starstead, Nümbrecht, Germany), and analyzed via UV-Vis spectroscopy (UV-2600, Shimadzu, Kyoto, Japan) using the 1.12 LabSolutions UV-Vis software. 

### 3.6. Antibiofilm Activity of MWCNT/PDMS Surfaces

#### 3.6.1. Bacterial Strains and Culture Conditions

*E. coli* CECT 434 and *S. aureus* ATCC 25923 were used as model bacteria to study the antibacterial activity of f-MWCNT_N/PDMS and f-MWCNT_N600/PDMS surfaces against Gram-negative and Gram-positive bacteria, respectively. Before each experiment, bacterial strains were collected from cryo-preserved aliquots of Luria–Bertani Broth (LB, Thermo Fisher Scientific, Waltham, MA, USA) containing 30% (*v*/*v*) glycerol and spread on Plate Count Agar (PCA, Merck KGaA, Darmstadt, Germany) plates, which were incubated overnight at 37 °C. Single colonies were inoculated in LB broth and incubated for 16 ± 2 h at 37 °C, 120 rpm. Subsequently, bacterial suspensions were centrifuged at 3772× *g*, 18 °C for 10 min (Eppendorf Centrifuge 5810R, Eppendorf, Hamburg, Germany) and pellets were resuspended in fresh LB medium to obtain final suspensions with an optical density at 610 nm of 0.15 for *E. coli* and 0.1 for *S. aureus*, which correspond to ~5.0 × 10^8^ colony-forming units per mL.

#### 3.6.2. Biofilm Formation Assay

To perform the antibiofilm assays, the control and nanocomposite surfaces were first sterilized by UV radiation for 30 min. The coupons were then placed on microplate wells (12-well microtiter plates, VWR International, Carnaxide, Portugal) and inoculated with 3 mL of bacterial suspension. Negative controls with 3 mL of LB medium were also prepared to evaluate the surface sterility throughout the experiments. Microtiter plates were incubated at 37 °C for 24 h under static conditions.

The antibiofilm performance of MWCNT/PDMS surfaces was assessed by determining the number of culturable cells. After 24 h of biofilm formation, coupons were removed from the microplate wells, submerged in 3 mL of sterile NaCl solution (8.5 g L^−1^), and vigorously vortexed for 3 min to obtain biofilm cell suspensions. These bacterial suspensions were then properly diluted in NaCl solution, spread on PCA, and incubated overnight at 37 °C for colony-forming units enumeration. At least three independent experiments were performed, each one with two technical replicates.

### 3.7. Characterization of MWCNT Antibacterial Mechanisms

The antibacterial effects of p-MWCNT, f-MWCNT_N, and f-MWCNT_N600 on *E. coli* and *S. aureus* cells were investigated using flow cytometry. Briefly, bacterial suspensions containing 5.0 × 10^8^ cells per milliliter were exposed to 5% (*w*/*v*) MWCNT for 24 h at 37 °C; non-treated cells were used as control. Bacteria were then harvested by centrifugation and the supernatant was collected for analysis [65].

Cell membrane potential and endogenous ROS production were evaluated by staining the cells with bis-(1,3-dibutylbarbituric acid) trimethine oxonol (DiBAC_4_(3); Sigma–Aldrich, Taufkirchen, Germany) and 2′,7′-dichlorofluorescein diacetate (DCFH-DA, Sigma–Aldrich, Taufkirchen, Germany), as previously described by Teixeira-Santos et al. [66]. After staining, bacteria were analyzed in a CytoFLEX flow cytometer model V0-B3-R1 (Beckman Coulter, Brea, CA, USA), using the CytExpert software (version 2.4.0.28). Samples were acquired at a flow rate of 10 µL min^−1^. The results were presented as the mean intensity of fluorescence (MIF) at FL1 (530 nm).

### 3.8. Cytotoxicity Assessment of MWCNT/PDMS Composites

To investigate the impact of MWCNT/PDMS composites on mammalian cell viability, PDMS and MWCNT/PDMS surfaces were tested using an immortalized human kidney proximal tubule (HK-2) cell line (ATCC, UK). Cells were first cultured in Dulbecco’s modified Eagle medium (DMEM; Invitrogen, Horsham, UK), supplemented with 10% fetal bovine serum (FBS; Lonza Bioscience, Cambridge, UK), and 50 µg mL^−1^ of penicillin and streptomycin (Life Technologies, Warrington, UK), at 37 °C in 5% CO_2_.

Surfaces were fixed to the bottom of 24-well plates using double-sided adhesive tape and sterilized for 1 h by ultraviolet radiation. Cells were seeded into the wells at a density of 25,000 cells per well and incubated for 24 and 48 h at 37 °C in 5% CO_2_. Following incubation, the culture medium was removed from the wells, and the viability of HK-2 cells was determined using the cell counting kit-8 (CCK-8; Sigma-Aldrich, Gillingham, UK). Briefly, 50 μL of the CCK-8 reagent along with 950 μL of DMEM were added to each well and incubated for 2 h at 37 °C in 5% CO_2_. The absorbance of each well was measured at 450 nm using a microplate reader (Thermo-Scientific Multiskan 60; Thermo-Fisher Scientific, Horsham, UK).

### 3.9. Statistical Analysis

Descriptive statistics were used to calculate the mean and standard deviation or error for surface roughness, the number of *E. coli* and *S. aureus* biofilm culturable cells, and the intensity of florescence of cells analyzed through flow cytometry. Data normality was assessed using the Kolmogorov–Smirnov and Shapiro–Wilk tests. Since the data were not normally distributed, the nonparametric Mann–Whitney test was applied to evaluate the differences in surface roughness and the number of *E. coli* and *S. aureus* biofilm culturable cells obtained for each surface. Significant differences were denoted as follows: * for *p* < 0.05, ** for *p* < 0.01, and *** for *p* < 0.001.

IBM SPSS Statistics version 26.0 for Windows (IBM SPSS, Inc., Chicago, IL, USA) was used to perform all of the statistical analyses.

## 4. Conclusions

This study provided new insights into the antibiofilm activity of MWCNTs with selective surface oxygen content against Gram-negative and Gram-positive bacteria. Among the tested samples, f-MWCNT/PDMS surfaces containing nitric acid-functionalized MWCNTs heated at 600 °C demonstrated the best performance in reducing the culturability of *E. coli* and *S. aureus* biofilms. The reduction in surface carboxyl groups and the redox activity of carbonyl groups has therefore been proven to play a preponderant role in MWCNT antimicrobial activity. Furthermore, these carbon materials seemed to exert their antimicrobial effect at the level of the cell membrane, inducing the production of ROS only in Gram-positive bacteria. Concerning biocompatibility, MWCNT-based surfaces did not have adverse effects on human cells, making them suitable for medical applications.

Altogether, these results indicate that f-MWCNT_N-based surfaces have the potential to be applied in the construction of medical devices and implants, although further studies are required to assess their long-term activity and stability.

## Figures and Tables

**Table 1:** Textural properties of pristine and modified MWCNTs.

Sample	S_BET_ (m^2^ g^−1^)	V_p P/P0=0.95_ (cm^3^ g^−1^)
p-MWCNT	196	0.419
f-MWCNT_N	210	0.408
f-MWCNT_N600	223	0.432

## Data Availability

The data presented in this study are available from the corresponding author upon request.

## Supplementary Materials

The following supporting information can be downloaded at: https://www.mdpi.com/article/10.3390/antibiotics12111620/s1, Figure S1: SEM images of (a) PDMS, (b) p-MWCNT/PDMS, (c) f-MWCNT_N/PDMS, and (d) f-MWCNT_N600/PDMS composites (magnification of 100×); Figure S2: SEM images of (a) p-MWCNT/PDMS and (b) f-MWCNT_N/PDMS cross-sections (magnification of 2000×); Figure S3: UV-Vis spectra of water after contact with PDMS, p-MWCNT/PDMS, f-MWCNT_N/PDMS, and f-MWCNT_N600/PDMS; Figure S4: Effect of MWCNT/PDMS composites on the viability of human kidney proximal tubule (HK-2) cells after 24 and 48 h of exposure. Significant differences between the viability of HK-2 cells exposed to PDMS and MWCNT/PDMS composites are denoted as *** (*p* < 0.001).
