# Supplementary material for: Antibiofilm Effect of Nitric Acid-Functionalized Carbon Nanotube-Based Surfaces against E. coli and S. aureus
Source: Antibiotics (Basel). 2023 Nov 11;12(11):1620. doi: 10.3390/antibiotics12111620 (PMC10668832; doi:10.3390/antibiotics12111620)
Supplement: Supplementary file 1 [file antibiotics-12-01620-s001.zip › antibiotics-2699703-supplementary.pdf]

Supplementary Material

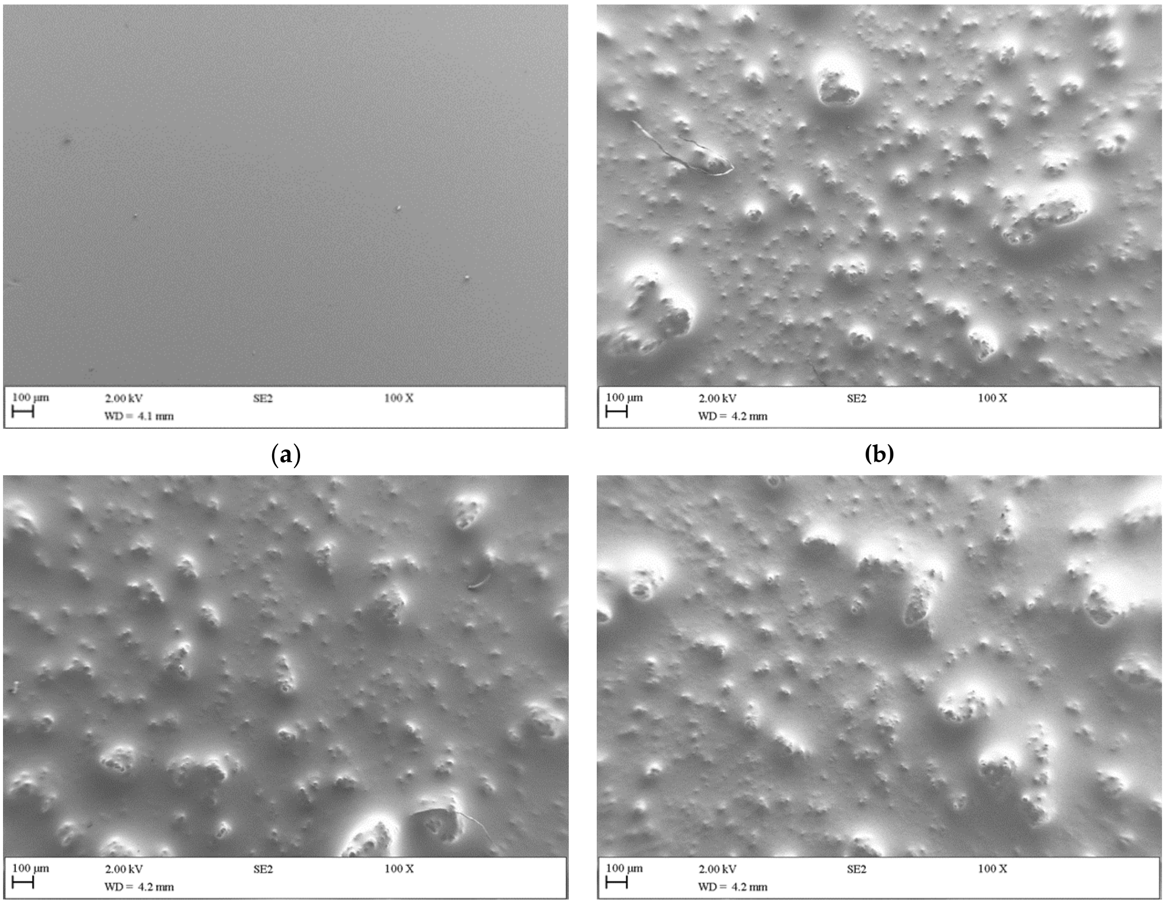

(**a**) (**b**)

(**c**) (**d**)

Figure S1. SEM images of (**a**) PDMS, (**b**) p-MWCNT/PDMS, (**c**) f-MWCNT_N/PDMS, and (**d**) f-MWCNT_N600/PDMS composites (magnification of 100×).

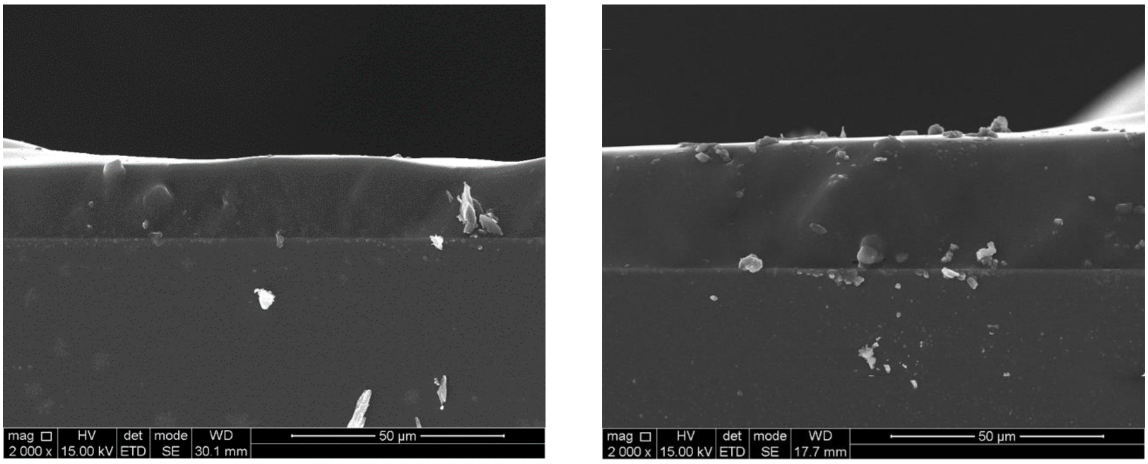

(**a**) (**b**)

Figure S2. SEM images of (**a**) p-MWCNT/PDMS and (**b**) f-MWCNT_N/PDMS cross-sections (magnification of 2000×).

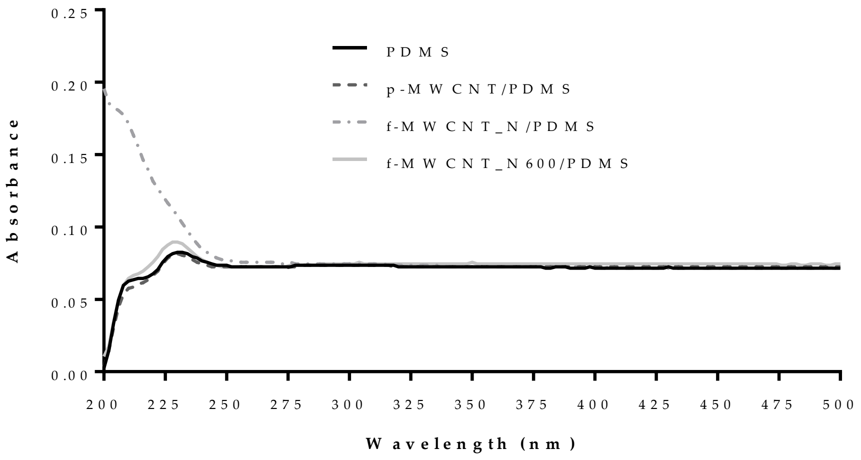

Figure S3. UV-Vis spectra of water after contact with PDMS, p-MWCNT/PDMS, f-MWCNT_N/PDMS, and f-MWCNT_N600/PDMS.

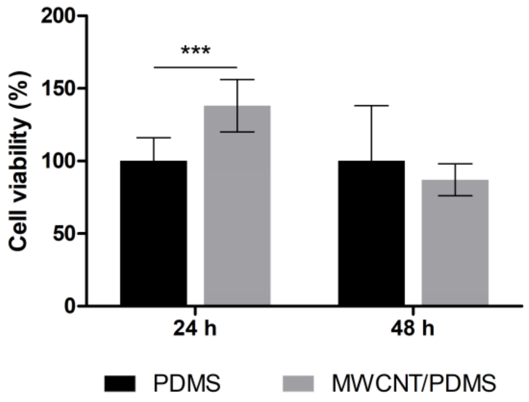

Figure S4. Effect of MWCNT/PDMS composites on the viability of human kidney proximal tubule (HK-2) cells after 24 and 48 h of exposure. Significant differences between the viability of HK-2 cells exposed to PDMS and MWCNT/PDMS composites are denoted as *** ($p < 0.001$).
